# PMLB v1.0: an open-source dataset collection for benchmarking machine learning methods

**DOI:** 10.1093/bioinformatics/btab727

**Published:** 2021-10-22

**Authors:** Joseph D Romano, Trang T Le, William La Cava, John T Gregg, Daniel J Goldberg, Praneel Chakraborty, Natasha L Ray, Daniel Himmelstein, Weixuan Fu, Jason H Moore

**Affiliations:** Institute for Biomedical Informatics, University of Pennsylvania, Philadelphia, PA 19104, USA; Center of Excellence in Environmental Toxicology, University of Pennsylvania, Philadelphia, PA 19104, USA; Institute for Biomedical Informatics, University of Pennsylvania, Philadelphia, PA 19104, USA; Institute for Biomedical Informatics, University of Pennsylvania, Philadelphia, PA 19104, USA; Institute for Biomedical Informatics, University of Pennsylvania, Philadelphia, PA 19104, USA; Department of Computer Science & Engineering, Washington University in St. Louis, St. Louis, MO 63130, USA; School of Arts and Sciences, University of Pennsylvania, Philadelphia, PA 19104, USA; Wharton School, University of Pennsylvania, Philadelphia, PA 19104, USA; Princeton Day School, Princeton, NJ 08540, USA; Related Sciences, Denver, CO 80220, USA; Department of Systems Pharmacology & Translational Therapeutics, University of Pennsylvania, Philadelphia, PA 19104, USA; Institute for Biomedical Informatics, University of Pennsylvania, Philadelphia, PA 19104, USA; Institute for Biomedical Informatics, University of Pennsylvania, Philadelphia, PA 19104, USA

## Abstract

**Motivation:**

Novel machine learning and statistical modeling studies rely on standardized comparisons to existing methods using well-studied benchmark datasets. Few tools exist that provide rapid access to many of these datasets through a standardized, user-friendly interface that integrates well with popular data science workflows.

**Results:**

This release of PMLB (Penn Machine Learning Benchmarks) provides the largest collection of diverse, public benchmark datasets for evaluating new machine learning and data science methods aggregated in one location. v1.0 introduces a number of critical improvements developed following discussions with the open-source community.

**Availability and implementation:**

PMLB is available at https://github.com/EpistasisLab/pmlb. Python and R interfaces for PMLB can be installed through the Python Package Index and Comprehensive R Archive Network, respectively.

## 1 Introduction

Benchmarking is a standard technique used for evaluating the strengths and weaknesses of machine learning (ML) algorithms with regard to different problem characteristics—namely, how well they perform on a group of well-studied benchmark datasets (Caruana and Niculescu-Mizil, 2006; Stallkamp *et al.*, 2012). Ideally, these datasets should have known measures of data quality (e.g. missing values, precision), previous results from other ML studies using the same dataset, and in the case of supervised learning, correct and unambiguous target values (i.e. dependent variables) used to calculate performance metrics for candidate models (Friedman *et al.*, 2001). In general, benchmarking involves assessing the performance of specific tools or protocols on a set of predefined tasks or datasets, and is used in many areas beyond evaluating ML models, such as software tools (Mangul *et al.*, 2019), research methods (Mitchell *et al.*, 2020; Weber *et al.*, 2019) and clinical practice guidelines (Nicolucci *et al.*, 2014), among others. Although benchmark datasets for ML are plentiful, they are often difficult to access, challenging to integrate with analyses of other datasets and prone to myriad data quality issues (Cortes *et al.*, 1995). PMLB (Penn Machine Learning Benchmarks) is a large, curated repository of open-source benchmark datasets that aims to solve these issues.

PMLB is typically used as a standalone package for the Python and R programming languages, and is available from standard package repositories. Users can select a classification or regression dataset from the collection, and then (in a single line of code) download the dataset, optionally save a local copy for future use, and load it into a data structure that is ready for use in popular machine learning libraries. Specific documentation with code examples is described Section 3.

The original prototype release of PMLB (v0.2) (Olson *et al.*, 2017) received positive feedback from the ML community, reflecting the pressing need for a collection of standardized datasets to evaluate models without intensive preprocessing and dataset curation. As the repository becomes more widely used, community members have requested new features such as additional information about the datasets, a standardized metadata schema, and new functions to find and select datasets given specific criteria, among others. In this Applications Note, we review PMLB’s core functionality and present new enhancements that facilitate fluid interactions with the repository, both from the perspective of database contributors and end-users (Table 1).

**Table 1.** Summary of PMLB datasets (with comparison to v0.2)

	PMLB v0.2	PMLB v1.0
Num. classification datasets	150	162
Num. regression datasets	0	255
Mean num. instances	20 865	42 860
Median num. instances	500	1066
Language interfaces	Python	Python; R
Miscellaneous tools	—	Interactive website
		Pandas Profiling reports
		Git LFS support
		API documentation
		Contributing guide
		Automatic dataset validation

To our knowledge, PMLB represents the largest publicly available collection of curated, ready-to-use ML benchmark datasets for classification and regression in existence. Competing ML dataset collections—such as the UCI Machine Learning Repository (Dua and Graff, 2017) or Kaggle Datasets—tend to contain a mixture of classification, regression and other datasets, with varying degrees of documentation/preprocessing and often inadequately characterized measures of data quality. Other, smaller collections of datasets—like Scikit-Learn’s datasets module (Pedregosa *et al.*, 2011)—can be well-documented and curated, but lack the breadth and scope of PMLB. PMLB aims to balance this tradeoff, a task which we approach through a combination of crowdsourcing datasets, automating the assessment of data quality, and utilizing appropriate third-party tools, such as GitHub’s continuous integration features, Pandas Profiling and Git Large File Store, as described in the following text.

## 2 Implementation

PMLB consists of three main components: (i) the collection of benchmark datasets, including metadata and associated documentation, (ii) a Python interface for easily accessing the datasets in the PMLB collection and (iii) an R interface providing similar functionality to the Python interface. PMLB synthesizes and standardizes hundreds of publicly available datasets from diverse sources such as the UCI ML repository and OpenML, enabling systematic assessment of ML methods using a single data interface. Copies of the individual datasets are stored in the GitHub repository using Git Large File Storage, and each dataset is accompanied by a user-provided set of metadata describing the dataset (including keywords that can be used to categorize datasets), as well as an automatically generated Pandas Profiling report that quantitatively describes various characteristics of each dataset.

### 2.1 New datasets with rich metadata

Since PMLB’s original release (v0.2) (Olson *et al.*, 2017), we have made substantial improvements in collecting new datasets. PMLB now includes benchmark datasets for regression problems (in addition to classification problems, which have been supported since earlier versions). Each dataset now includes a metadata.yaml file containing general descriptive information, including the original web address of the dataset, a text description of its purpose, any associated publications, keywords and descriptions of individual features and their coding schema, among others. Metadata files are supported by a standardized format that is formalized using JSON-Schema (version draft-07) (Pezoa *et al.*, 2016). Upcoming releases of PMLB improve upon the automated validation of datasets and metadata files to simplify contributions and maintain data accuracy.

### 2.2 User-friendly interfaces

The new version of PMLB includes a contribution guide with step-by-step instructions on how to add new datasets, edit existing datasets or improve the Python or R interfaces. When a user adds a new dataset, summary statistics are automatically computed, a profiling report is generated (see below), a corresponding metadata template is created. Once changes are approved, PMLB’s list of available datasets is automatically updated.

**Fig. 1.**
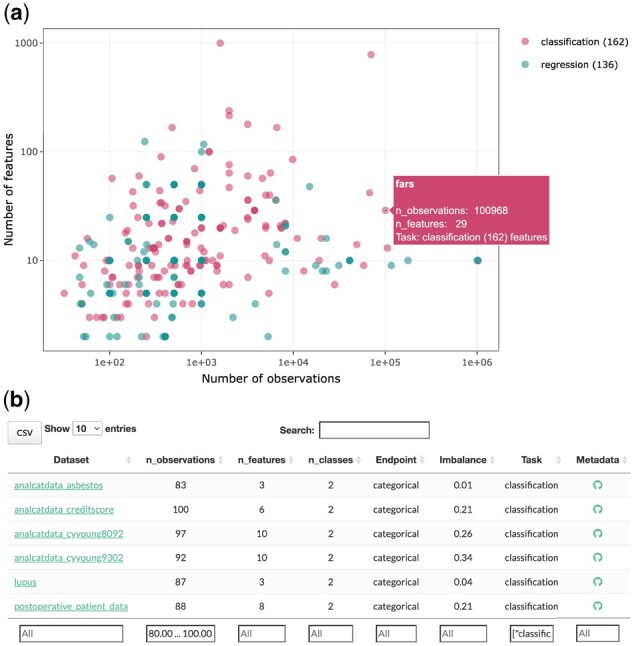
Database search features on PMLB’s website. (**a**) Interactive scatterplot of databases in PMLB, showing number of features and number of observations in each dataset, as well as whether it is a regression or classification dataset. (**b**) Responsive table of PMLB databases. Users can sort on any columns’ values or filter based on ranges of values. Clicking on any dataset name will bring the user to the Pandas Profiling report for that dataset

On PMLB’s homepage, users can now browse, sort, filter and search for datasets using a responsive table that includes summary statistics (Fig.  1). In addition to the existing Python interface for PMLB, we have included an R library for interacting with PMLB. The R library includes a number of detailed ‘vignette’ documents to help new users learn how to use the software. The website includes API reference guides detailing all user-facing functions and variables in PMLB’s Python and R libraries.

### 2.3 Pandas profiling reports

We generate summary statistic and metadata reports for each dataset using pandas-profiling. These reports provide detailed quantitative descriptions of each dataset, including correlation structures between features and flagging of duplicate and missing values. Browsing the reports allows users and contributors to rapidly assess dataset quality and make any necessary changes. For example, if a feature is flagged as containing a single value repeated across all samples, it is likely that the feature is uninformative and should be removed from ML analyses. Profiling reports can be accessed either by navigating to the dataset’s directory in the PMLB code repository, or by clicking the dataset name in the interactive dataset browser on the PMLB website.

## 3 Availability

PMLB is publicly available, open-source and released under the MIT license. User-friendly interfaces are available for the Python and R programming languages, and can be installed via the Python Package Index (PyPI) and the Comprehensive R Archive Network (CRAN), respectively. The source code repository for PMLB is maintained at https://github.com/EpistasisLab/pmlb, and documentation for PMLB is provided at https://epistasislab.github.io/pmlb.
